# Targeting lipid droplets and lipid droplet-associated proteins: a new perspective on natural compounds against metabolic diseases

**DOI:** 10.1186/s13020-024-00988-w

**Published:** 2024-09-04

**Authors:** Xinyue Jiang, Hongzhan Wang, Kexin Nie, Yang Gao, Shen Chen, Yueheng Tang, Zhi Wang, Hao Su, Hui Dong

**Affiliations:** 1 Institute of Integrated Traditional Chinese and Western Medicine, Tongji Hospital, Tongji Medical College, Huazhong University of Science and Technology, Wuhan, China; 2 Department of Integrated Traditional Chinese and Western Medicine, Tongji Hospital, Tongji Medical College, Huazhong University of Science and Technology, Wuhan, China; 3 Department of Rehabilitation Medicine, Tongji Hospital, Tongji Medical College, Huazhong University of Science and Technology, Wuhan, China

**Keywords:** Lipid droplet, Lipid droplet-associated proteins, Metabolic diseases, Natural compounds

## Abstract

**Background:**

Lipid droplet (LD) is a metabolically active organelle, which changes dynamically with the metabolic state and energy requirements of cells. Proteins that either insert into the LD phospholipid monolayer or are present in the cytoplasm, playing a crucial role in lipid homeostasis and signaling regulation, are known as LD-associated proteins.

**Methods:**

The keywords “lipid droplets” and “metabolic diseases” were used to obtain literature on LD metabolism and pathological mechanism. After searching databases including Scopus, OVID, Web of Science, and PubMed from 2013 to 2024 using terms like “lipid droplets”, “lipid droplet-associated proteins”, “fatty liver disease”, “diabetes”, “diabetic kidney disease”, “obesity”, “atherosclerosis”, “hyperlipidemia”, “natural drug monomers” and “natural compounds”, the most common natural compounds were identified in about 954 articles. Eventually, a total of 91 studies of 10 natural compounds reporting in vitro or in vivo studies were refined and summarized.

**Results:**

The most frequently used natural compounds include Berberine, Mangostin, Capsaicin, Caffeine, Genistein, Epigallocatechin-3-gallate, Chlorogenic acid, Betaine, Ginsenoside, Resveratrol. These natural compounds interact with LD-associated proteins and help ameliorate abnormal LDs in various metabolic diseases.

**Conclusion:**

Natural compounds involved in the regulation of LDs and LD-associated proteins hold promise for treating metabolic diseases. Further research into these interactions may lead to new therapeutic applications.

**Graphical Abstract:**

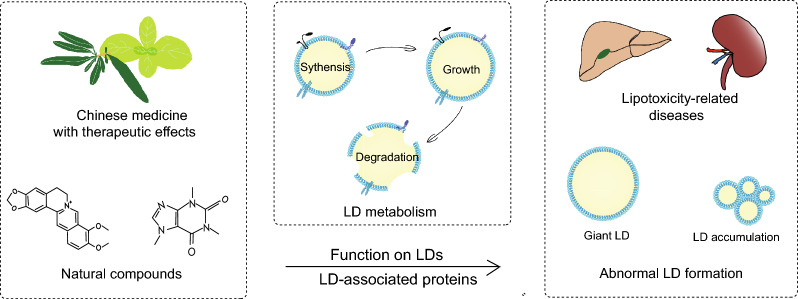

## Introduction

The prevalence of metabolic diseases, including obesity, dyslipidemia, type 2 diabetes mellitus (T2DM), and non-alcoholic fatty liver disease (NAFLD), is escalating yearly, posing a serious threat to human health [1]. According to the latest epidemiology, overweight rates in adults reached 42.4% and 59% in the United States and Europe, respectively [2]. The global incidence of NAFLD has reached 32.4% by 2021 [3]. The optimal treatment for these diseases involves improving lifestyle and dietary habits, yet the outcome is poor due to a lack of patient adherence [4].

As an active organelle, lipid droplet (LD) varies dynamically with the metabolic state and energy requirements of cells. Early research established that LDs are crucial for lipid metabolism, with abnormalities often leading to lipotoxicity-related diseases, like diabetes, and fatty liver diseases [5]. LD-associated proteins, also known as LD proteins, are decorative proteins of phospholipid monolayers, including lateral proteins and gross membrane proteins which employ a single topology [6]. Primary studies of LD-associated proteins referred to classical protein families like perilipins (PLINs), which were found to prevent lipolysis of LDs [7–10]. Subsequently, more molecules are gradually being recognized as LD-associated proteins, like Cell death-induced DNA fragmentation factor 45-like effector (CIDE), hypoxia inducible gene 2 (HIG2), etc. [11, 12]. LD-associated proteins are involved in various physiological activities such as LD biogenesis, growth, fusion, and lipolysis, thus affecting lipid homeostasis [13]. Therefore, targeting the regulation of LDs and LD-associated proteins may offer novel approaches for the therapy [14].

Massive basic or clinical studies have confirmed that traditional Chinese medicine could remarkably confront metabolic disorders by improving lipid metabolism. However, traditional Chinese medicine have complex components and their pharmacological mechanism have not been thoroughly elucidated, resulting in limited application. Moreover, the potential role in regulating LDs remains unknown. The keywords “lipid droplets” and “metabolic diseases” were used to obtain literature on LD metabolism and pathological mechanisms. After searching databases including Scopus, OVID, Web of Science, and PubMed from 2013 to 2024 using terms like “lipid droplets”, “lipid droplet-associated proteins”, “fatty liver disease”, “diabetes”, “diabetic kidney disease”, “obesity”, “atherosclerosis”, “hyperlipidemia”, “natural drug monomers” and “natural compounds”, we identified the most common natural compounds in about 954 articles. Eventually, a total of 91 studies of 10 natural compounds reporting in vitro or in vivo studies were refined and summarized to elucidate the mechanism by which natural compounds affect LDs and LD-associated proteins. We aim to provide a reference for further research on the regulatory mechanism of natural compounds and their effective management of metabolic diseases.

## Connection between LD dynamics and LD-associated proteins

LD primarily consists of a core formed by triacylglycerol (TAG) and cholesterol ester (CE), which are enveloped by an amphiphilic phospholipid monolayer containing numerous embedded proteins on the surface [15]. In addition to storing and regulating lipid utilization [16], LD serves as a reservoir for proteins [17]. The proteomics of LD has revealed over 200 protein components, some of them fixate only in LDs, while others are also present in other subcellular compartments [18]. As a metabolically active organelle, the size, morphology, number, lipid composition and protein composition of LD can change according to the metabolic situation of the organism. The dynamics of LD include LD biogenesis, expansion, fusion, degradation, and contacts with other cellular organelles [19]. The orderly unfolding of these processes depends on LD-associated proteins.

### LD-associated proteins

Various LD-associated proteins can be generally classified according to their functions as follows: enzymes that catalyze lipid metabolism, histones that bind nucleic acids to LDs, ribosomal proteins that exist in protein synthesis in prokaryotes, proteins from protein degradation, proteins with signaling capabilities, membrane transport proteins that interact with other organelles, dynamics related proteins such as microtubules and skeletal proteins responsible for movement, LD resident proteins that are directly targeted to LDs with subcellular structural characteristics, etc. [20].

Protein targeting occurs in multiple biological processes in LD including LD biogenesis, growth, degradation, etc. Based on their targeting pathways, LD-associated proteins can be subdivided into two classes. The first class, “ERTELLED” (classical class I), consists of proteins inserted into the endoplasmic reticulum (ER) membrane that relocate to the LD surface through ER-LD contact. The second class, “CYTELLED” (classical class II), comprises proteins translated in the cytoplasm that directly target the LD surface [21, 22].

ERTELLED proteins generally comprise hydrophobic membrane-embedded sequences that acquire a hairpin configuration in the ER bilayer. This class includes esteryol coenzyme A synthase (e.g. Acyl-CoA synthetase long-chain family member 3 (ACSL3), ACSL5) [23], triglyceride lipase (e.g. adipose triglyceride lipase/Patatin-like phospholipase domain-containing protein 2 (ATGL/PNPLA2) [24], acyltransferases (e.g. GPAT4, LPCAT) [25], LD-associated hydrolase [26], LD assembly factor 1 (LDAF1, also known as TMEM159) [27], and UBX structural domain-containing protein 8 (UBXD8) [28], etc.

CYTELLED proteins are soluble proteins with several LD-binding amphipathic helix motifs, including the PLIN family, comparative gene identification-58 (CGI-58, also known as ABHD5) and Phosphocholine cytidylyltransferase (PCYT or CCT) [29–31]. Among these, the PLINs are the main LD-associated proteins [32]. PLIN1 and PLIN4 are mainly expressed in White adipose tissue (WAT). PLIN5 is generally expressed in the liver, heart, BAT and skeletal muscle. Meanwhile, PLIN2 and PLIN3 are widely expressed in various tissues [33].

### LD Biogenesis

Present studies supposed that the synthesis of neutral lipids plays a major part in LD biogenesis. The neutral lipids synthesis from activated fatty acids (FAs) represents the first step of LD biogenesis. If FA esters to diacylglycerol (DAG), it is further catalyzed by diacylglycerol acyltransferases to produce TAG. Similarly, if FA esterifies to sterols (e.g. cholesterol), they are catalyzed by cholesterol o -acyltransferases to CE afterward [34].

**Fig. 1:**
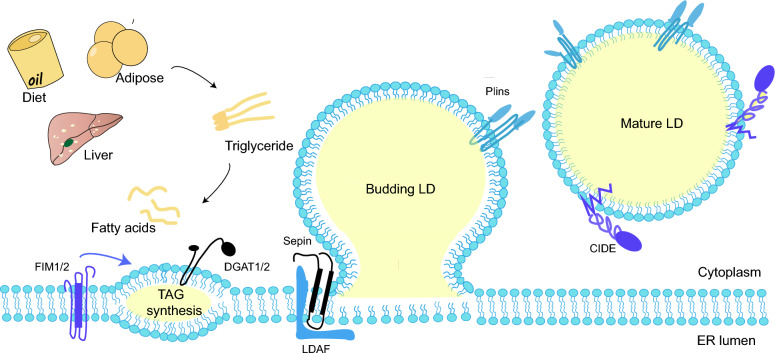
LD biogenesis. Activated fatty acids produce neutral lipids in TAG synthesis. Wrapped by the phospholipid molecule shell deriving from the ER leaflets, the neutral lipids sprout into oil lenses and grow larger. When the superficial tension attains a certain level, the budding LD flips form a mature LD

ER-LD contact is known to make sense for LD formation [35]. Seipin, an ER protein that mediates ER-LD contacts, determines the site where LDs form in the ER and interacts with LDAF1 to form the LDAF1-Seipin complex [36]. The complex co-purifies with TAG, followed by TAG elution by membrane phospholipids, forming LDs [37]. After LD formation, LDAF1 splits from Seipin, turning to the LD surface. While lacking the complex, only high levels of TAG (4% or above) can facilitate LD formation [38]. Seipin also works as a scaffolding protein to recruit enzymes like 1-acylglycerol-3-phosphate O-acyltransferase 2 (AGPAT2) and lipoprotein 1 (Lipin-1), and restrains palmitic acid (PA) levels by reducing Glycerol-3-phosphate acyltransferase (GPAT) [39, 40] (Fig. 1).

Nucleus LDs (nLDs) are commonly found in cultured cells of liver and hepatic origin [41, 42], with a higher ratio of CE and TAG compared to cytoplasmic LDs (cLDs). Interestingly, although Seipin is an essential protein for cLD formation, the knockdown of Seipin may increase the amount of nLDs in the experimental strains instead [43].

### LD growth

**Fig. 2:**
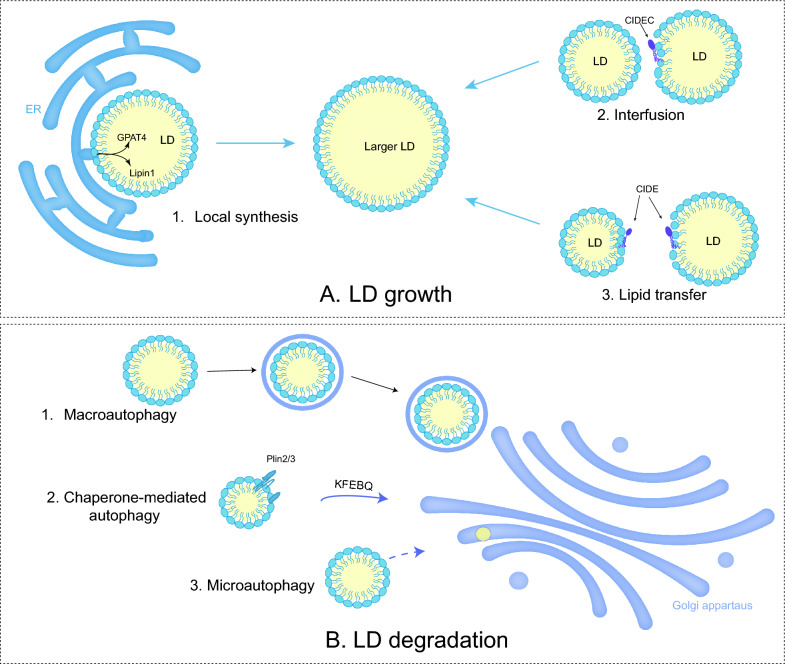
LD growth and LD degradation. **A** 1. When LDs contact with ER, adipogenic enzymes catalyze the TAG synthesis. 2&3. Both LD fusion and lipid transfer between LDs render the growth. **B** 1. Macroautophage is the process of formation of autophagosome by double-membrane vacuoles before fusion with lysosomes. 2. Chaperon-mediated autophagy (CMA) refers to lysosomal selective capture of LDs containing the KFERQ motif. 3. Direct lysosomal degradation of LDs is microautophagy

After initial biogenesis, LD tends to expand by fusion between LDs and local lipid synthesis (Fig. 2A). LD fusion occurs through LD-LD coalescence or LD-LD lipid transfer [44]. LD coalescence occurs when the stability of phospholipid monomolecular membrane of LDs decreases due to a reduction in phosphatidylcholine (PC) or the aggregation of fusogenic PA [31]. Phosphocholine cytidylyltransferase (PCYT or CCT) is a key rate-limiting enzyme in the Kennedy pathway of PC synthesis. Among them, PCYT1A (CCT1) can be transported directly from the cytoplasm to the surface of LDs [45]. The knockdown of PCYT1A results in increased production of LDs, while its activation promotes the maintenance of LD homeostasis [31, 46].

CIDE can moderate the formulation of oversized LDs in adipocytes by mediating LD-LD lipid transfer. CIDEs locate to LDs through a carboxy-terminal amphiphilic helix to generate a dimer. The dimer diffuses on the LD surface and engages with another LD containing CIDE. The presence of CIDE on both LD surfaces promotes the formation of stable trans-organelle oligomers, which provide channels for lipid transfer [47]. This process allows neutral lipids to migrate from smaller LDs to larger LDs, while protein transfer does not occur [48]. In human hepatocytes, CIDEB promotes TAG transfer, but this catalytic role relies on the expression of CIDEC or CIDEA [49].

In addition, neutral lipid synthase-mediated local lipid synthesis is involved in the growth of LDs. The previously mentioned membrane bridge in LD-ER contact enables adipogenic enzymes like diacylglycerol acyltransferase 2 (DGAT2) and GPAT4 from ER to the LD surface, catalyzing the local synthesis of neutral lipids [50]. Lipin-1 (also known as PAH1) with phosphatidic acid phosphatase (PAP) activity, co-localizing with other proteins like PLIN2 and PLIN3, catalyzes PA into DAG [51]. Eventually, LD formation arises at the ER-LD interface [52].

### Contacts with other organelles

Recent research has revealed that LDs inevitably come into contact with other organelles when performing biological functions, through the fusion with phospholipid monolayers and variations of LD-associated proteins [53, 54]. LD-ER contact triggers LD generation and LD-associated protein targeting; LD-LD contact causes LD enlargement; LD-nuclear membrane contact lays the basis for nLD formation; LD-lysosome contact provides conditions for autophagy. In addition, LDs also seem to contact with mitochondria and peroxisome.

During nutrient deprivation, LD-mitochondrial contact function as sites of lipogenesis or catabolism [55, 56]. Although the specifies of LD-mitochondrial haven’t been clarified yet, studies have found that PLIN5 overexpression in BAT to induces mitochondrial recruitment at the LD periphery [57], and PLIN1 interacts with mitochondrial protein 2 [58]. A recent review summarized LD-associated protein interactions that mediate LD-mitochondrial contact: PLIN5-fatty acid transporter protein (FATP4) and Mitofusin2 (MFN2)-Heat shock cognate 71 kDa protein (HSC70)-PLIN1 for fatty acid oxidation; ADP-ribosylation factor related protein 1 (ARFRP1)-Synaptosome-associated protein 23 (SNAP23) and mitoguardin 2 (MIGA2)-Vacuolar Protein Sorting 13D (VPS13D) for LD growth [59].

Similar to the function of mitochondria, the peroxisome is the only setting of β -oxidation in yeast, along with branched-chain fatty acids and β -oxidation of very long-chain fatty acids in humans [60]. The molecular basis for the fusion between the outer layer of peroxisomal phospholipid leaflets and the LD membrane still requires clarification [61].

### LD degradation

LD degradation frequently occurs, though not exclusively, following the completion of biological processes. The most common form of degradation for LDs is lipolysis, which requires ATGL /PNPLA2 and its activator CGI-58, hormone-sensitive triglyceride lipase (HSL), and monoacylglycerol lipase (MAGL) [62–64], etc. Moreover, ATGL is activated via the patatin structural domain interacting with CGI-58, as well as inhibited by CIDEC. During nutrient deprivation, LDs can also degrade by lipophagy, a selective autophagic procedure that transports part or the entire LDs to lysosomes, undergoing bulk degradation by hydrolytic enzymes [65].

The PLIN family, governed by sterol regulatory element binding protein (SREBP), liver X receptor (LXR) and peroxisome proliferators-activated receptor (PPAR), affects LD degradation by regulating the efficiency of lipase entry to the surface of LDs [66]. For instance, chaperone-mediated autophagy (CMA) degradation of PLIN2/3 promotes the entry of ATGL and autophagy proteins into the surface of LDs. The Rab proteins, consisting of around 70 small GTPases, modulate cytoskeletal motility and are also involved in the autophagic process [67]. Rab7 regulates the interaction between lysosomes and autophagosomes through members of the homotypic fusion and protein sorting (HOPS) tethering complex and soluble NSF attachment protein receptor (SNARE) proteins [68] (Fig. 2B).

## The role of LDs in relevant diseases

**Fig. 3:**
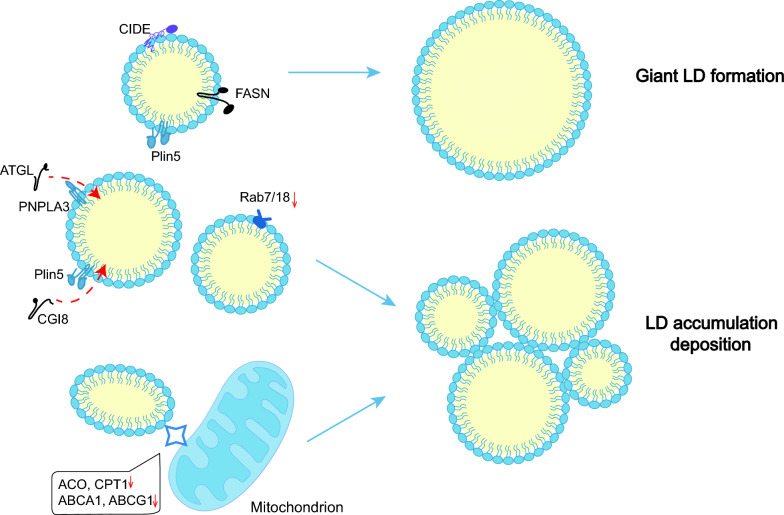
Abnormal LD formation in metabolism-related diseases. The FASN-mediated lipid synthesis system and CIDE-mediated LD fusion promote the generation of giant LDs. Moreover, the expression of ATGL, CGI8, Rab7/18, ACOX, CPT1, ABCA1, and ABCG1 is downregulated, inhibiting lipolysis and β-oxidation, resulting in LD deposition

LDs play a vital part in lipid metabolism and signaling pathways. Abnormalities of LD-associated proteins leads to dysregulation of LD dynamics, resulting in significant disorders (Fig. 3). The available evidence indicates that LDs and LD-associated proteins are closely related to metabolic diseases.

### Metabolic diseases

NAFLD, is a spectrum of chronic liver diseases that includes simple hepatic steatosis, nonalcoholic steatohepatitis, fibrosis and cirrhosis [69]. During steatosis, excessive triglycerides are stored in LDs [70]. The continuous generation of LDs, frequent LD fusion and impaired LD autophage contribute to LD accumulation [71, 72]. Furthermore, extremely large LDs cluster in hepatocytes [73].

In mouse models, CIDEC, CIDEA and PLIN2 are highly upregulated in hepatocytes [74, 75], while the same changes of CIDEC occur in patients [76]. PLIN2 may display an anti-lipolytic impact by disrupting the action of ATGL on LDs [77]. Knockdown of PLIN5 in high-fat-diet (HFD) mice results in reduced steatosis and fibrosis in the liver, suppression of SREBP1 and its downstream fatty acid synthase (FASN),and inactivation of adenosine 5’-monophosphate-activated protein kinase (AMPK) [78]. Besides, PNPLA3 (I148M) accumulation on the LD surface may segregate CGI58, thus promoting lipolysis [79, 80]. As for the Rab family, both Rab18, which has a regulatory role in lipolysis, and Rab7, which is related to autophagy are decreased in steatotic livers [81]. Serum levels of very low-density lipoprotein (VLDL) and TAG from VLDL are reduced in mice with CIDEB loss, while TAG levels in liver are increased [82].

Diabetes is also closely related to the homeostasis of LDs. Dai et al. found that mice transplanted with human islets developed more LDs in islet cells after a HFD [83], while hypertrophic LDs stack in adipocytes [59]. Hyperglycemia induce a significant fraction of lipophagy, ultimately resulting in LD accumulation and inflammation [84]. Similarly, renal biopsies from patients with diabetic nephropathy (DN) showed increased lipid deposition and LDs [85], which may be a compensatory protective pathway against lipotoxicity [86]. Resulting from increased PLIN2, decreased autophagic flux and excessive PPARδ activated by lysophosphatidylcholine, LD accumulation contributes to the decline in rapid renal function in DN [87].

Insulin secretion may be regulated by LD degradation. Lipolysis-generated FA promotes insulin secretion by prompting the PPARδ pathway and adenosine 5′-triphosphate production in mitochondria [88]. Local FA activates cell surface receptors (e.g. FAR1) and enhances insulin secretion [88]. Meanwhile, lipolysis-generated 1-monoacylglycerols (1-MAG) also stimulates insulin granule cytosolic action [89]. Unlike healthy people, patients with T2DM fail to undergo lipolysis induced by glucose for a currently unknown mechanism. However, present studies have confirmed that when the key enzymes for lipolysis (e.g. ATGL) become defective, palmitoylation of insulin synthesis protein 1a (STX1a) decreases, accelerating the degradation of STX1a and lowering insulin secretion [90].

### Renal diseases

When acute kidney injury (AKI) occurs, there is a deficiency of AMPKα with LD accumulation in the renal tubules [91]. In the plasma of patients with primary focal segmental glomerulosclerosis, the circulating permeability factor induces LD accumulation and overexpression of PLIN2 (a marker of renal fibrosis) [92, 93]. Fibrosis in the renal interstitium features increases LDs in proximal tubular cells, and the expression of PPARα, carnitine palmitoyltransferases 1 (CPT1), acyl-Coenzyme A oxidase 1 (ACOX1), PPARγ coactivator 1α (PGC-1α), and fatty acid binding protein 4 (FABP4) elevates [94].

AKI is a substantial risk for the occurrence and development of chronic kidney disease (CKD) [95]. In CKD models, the expression levels of CPT1, ACOX1, and L-FABP decrease significantly [96, 97]. Instead, the expression levels of LDL receptor (LDLR) and SREBP-2 increase significantly [96, 97]. CPT1, ACOX1, and L-FABP are the crucial proteins in fatty acid β-oxidation pathway, while LDLR and SREBP-2 are associated with cholesterol uptake. Reduced catabolism and excessive sterol intake result in enhanced neutral lipid content from LDs and eventually lead to the formation of giant LDs [98, 99].

### Cardiovascular diseases

In atherosclerosis (AS), lipids, primarily CE, increase the phagocytic load of macrophages, leading to the formation of more foam cells. Compared to normal areas of arteries at the same level, the high expression of PLIN2 in plaques induces the overexpression of pro-inflammatory cytokines [100, 101]. Additionally, PLIN2 aggravates the accumulation of CE in macrophages by restricting cholesterol efflux [102]. CIDEB-knockout mice show reduced plasma LDL and cholesterol levels, but increased liver cholesterol, which may result from the increased expression of LDLR and Acyl coenzyme A-cholesterol acyltransferase (ACAT1) [103]. Although HSL has a broad substrate specificity, it vanishes in human AS progression [104].

Clinical studies have shown that PLIN5 increased during heart attacks, similar to the high-sensitivity cardiac troponin I, thus may serve as a biomarker [105]. In addition, PLIN5, previously mentioned, functions in mediating LD-mitochondria contact, may provide new avenues for the therapy of myocardial ischemia–reperfusion injury (IRI). In the mouse models, inhibition of PLIN5 can promote cardiomyocyte proliferation and activate the PPAR signaling pathway, reduce the expression of PPARγ but raise the expression of PPARα [106]. Above all, increased PLINs may be the reason for more LDs, while various lipotoxic intermediates leading to excessive deposition of LDs in vesicles in the myocardium and eventually causing cardiac steatosis [107].

### Cancer

Lipid mobilization of LDs provides additional energy for drastically proliferating cancer cells: the FAs from core catabolism enter the mitochondria for oxidative energy supply [108]. LDs can also ensure cancer cell survival by reducing lipotoxicity via storing excess lipids in the inflammatory microenvironment [109]. ACSL4 can increase the contents of TAG and LDs in hepatocellular carcinoma cells [110]. ACSL3, which is required for exogenous fatty acid lipogenesis, expresses more to induce LD accumulation in renal cell carcinoma [111–113]. The synthesis of TAG, along with CIDEC and PLIN3, appears to be highly expressed with the decrease of CIDEB. The lipogenic proteins like ACSL4 and ACSL4 are upregulated, while CIDEB involved in TAG transfer is downregulated, which ultimately results in LD accumulation. What’s more, LDs may remove misfolded proteins by regulating fatty acids, the mechanism of which remains to be investigated [114].

LD-associated proteins have diagnostic, therapeutic and prognostic values in cancer [115], as exemplified by PLINs. PLIN2 has enhanced expression in urological cancers [116], and also has reference in the diagnosis of breast cancer staging and colorectal cancer [117, 118]. PLIN1 has potential to inhibit breast cancer [119]. PLIN4 overexpresses in adriamycin-resistant cells, which can be targeted to eliminate chemoresistance [119].

### Other diseases

Congenital lipodystrophy (CGI) is a group of heterogeneous disorders defined by a particular lack of fatty tissue with ectopic steatosis, dyslipidemia, and insulin resistance (IR) [120]. The supplementation of leptin may alleviate related symptoms. CGI is a scarce autosomal recalculant disease, relevant to the gene deletions of AGPAT2 (BSCL1), Seipin [121].

Some common pathogens usually metabolize by means of LDs in host cells. The hepatitis B virus invokes hepatic steatosis in the mouse model by targeting its core protein to LDs and enhancing LD-ER contact. This is achieved through NS5A binding to Rab18, which promotes LD generation and LD-associated protein targeting [122, 123]. Knockdown of PLIN3 attenuates this steatosis [124]. In addition to mediating pathogen infection, LDs also play an immunological role. For instance, the translation of viperin (an innate immune protein) posts on LDs [125].

## Links between natural compounds and LDs

Studies on natural compounds associated with lipid regulation have mainly focused on signaling pathways like AMPK, mammalian target of rapamycin (mTOR) and PPAR. Some downstream targets of these signaling pathways, which are involved in lipid metabolism, are also discovered to be localized on LDs. These targets including GPAT4, DGAT1, DGAT2, CPT1, and ATGL, are regarded as LD-associated proteins [53, 54, 126, 127]. Exploring the connection between natural compounds and LDs is of great significance for their clinical use and further development. Based on the current studies, we have summarized the mechanisms of lipid-lowering and regulation of LD homeostasis by natural compounds from the perspective of LD-associated proteins. We aim to provide new references for the clinical utilization and development of theses natural compounds (Table 1).

**Table 1:** Natural compounds acting on LD-associated proteins

Natural compounds	Model	Positive control	Usage	Dosage & Duration	Functions upregulate downregulate		Refs	Changes to LD(Lipid)
BBR	C57BL/6 J mice	–	I.G	300 mg/kg/day for 4 wk	–	Liver: SCD1, FASN	Zhu X et al. [129]	Content↓ Number↓ Surface area↓
	C57BL/6 J mice	ROT	P.O	1.4 g/kg mixed with HFD for 5mons	Liver: pACC	Liver: SCD1, FABP1, CD36, CPT1a,	Yu M et al. [130]	
	Black sea bream	–	P.O	50 mg/kg mixed with HLD for 8 wk[3 times daily]	Liver: LPL, HSL CPT1	Liver: ACCα, 6-PGD	Wang L et al. [131]	
	ApoE-/-mice	Atorvastatine	P.O	25, 50 mg/kg mixed with HFD for 6 wk	–	Endothelial: ASCL4	(Yang Hong et al., 2024) [238]	
	3T3-L1 cells	–	–	25, 50, 100 μM for 8 days	HSL, ATGL, pACC, ACSL1	FASN, FABP4	Sim MO et al. [137]	
	HepG1 cell	–	-	20 μM for 24 h	–	SCD1, FASN	Zhu X et al. [129]	
	Hepatocytes	–		1,2,4 μmol/L for 24 h	–	SCD1, CD36, CPT1a	Yu M et al. [130]	
	Cancer cells	–	–	From 6.25 to 50 μM for 24, 48, 72 h	–	FASN	Liu YX et al. [133]	
	Wistar rats	–	P.O	50, 100, 150 mg/kg BW/d mixed with HLD for 6 wk	Liver: CD36, CPT1, pACC	Liver: DGAT2	Niu Y et al. [140]	TG↓ FFA↓
	Syrian hamsters	–	P.O	50, 150 mg/kg BW mixed with HFD for 8 wk	Liver: CD36, CPT1 Muscle: CPT1	Liver: ACC	Guo F et al. [141]	
	KK-Ay mice	–	I.G	100,200 mg/kg/d for 4 wk	Liver: CPT1	Liver: FASN	Li J et al. [144]	
	Male Wistar rats	–	I.G	100,200 mg/kg/d for 8 wk	Liver: CD36	Liver: DGAT2	Zhang et al. [243]	
	Male SD rats	–	I.G	50 mg/kg/d for 30 d	–	(pre)-adipocytes: SCD1	(Ying-Hao Hu et al., 2021) [239]	
MGF	HepG2 cells		–	12.5, 25, 50, 100 µmol/L for 24 h	CD36, CPT1, pACC	DGAT2	Niu Y et al. [140]	
	HepG2 Cells	–	–	12.5, 25, 50 μM for 24 h	CPT1, CD36	–	Zhang Q et al. [140]	
	HepG2 Cells	–	–	metabolites of MGF	Liver: ACC, ATGL, CPT1, HSL	–	Li J et al. [144]	
	HT29 cancer cells	–	–	400 µM blended into 0.2 mM FFA for 24 h	–	CPT1	Rodriguez-Gonzalez JC et al. [143]	
	3T3-L1 cells	–	–	Water extracts	–	LPL	Baek J et al. [147]	
CAP	C57BL/6 J obese mice	–	Cream onto shaved abdominal skin	100 mg/d for 8wk	Liver: CPT1, CD36	Liver: ACC, FAS	Shin MK et al. [155]	Formation↓ Size↓ Surface area↓
	C57BL/6 J mice	–	–	0.01% mixed with HFD for 24 wk	Liver: tHSL, pHSL, CPT1	–	Li Q et al. [152]	
	Mesenchymal stem cells(mMSC)	–	–	10, 50, 100, 200 μM for 6 days	–	FABP4, SCD	Jeong JY et al. [153]	
	3T3-L1&X9 cells	Norepinephrine	–	1 μM for 24 h	PLIN1, CIDEA, CIDEC	–	Montanari T et al. [154]	
	Visceral adipose tissues	­	–	0.01% mixed with standard laboratory chow for 5 mon	HSL	–	Chen J et al. [150]	
	HepG2 Cells	–	–	200 µM for 24 h	PGC-1α, CD36	ACC	Bort A et al. [151]	
CAF	Zebrafish	–	P.O	1, 2.5, 5, 8% mixed with larval food for 20 days	Liver: ACO	Liver: (FAT)/CD36, UCP2, ACC1	Zheng X et al. [160]	Content↓ Number↓ Size↓ Redistributed evenly
	Wistar rats	­–	P.O	20, 30 mg/kg/day combined with HFD for 8 wk	Liver: CPT1	Liver: FAS, ACC	Helal MG et al. [161]	
	mMSCs	–	–	0.1, 1 mM for 7 days	–	LPL	Su SH et al. [159]	
	mMSCs	–	–	1 mM for 7 day	FABP4, UCP1, PGC-1α	–	Velickovic K et al. [163]	
GEN	SD rats	–	P.O	50 mg/kg diet for 90 days	–	Liver: FSP27	Xiao CW et al. [169]	Formation↓ Accumulation↓
	C57BL/6 J mice	–	P.O	1, 2, 4 g/kg mixed with HFD for 12wk	–	Liver: ACC2	Kim MH et al. [167]	
	Hens	–	P.O	40, 400 mg/kg mixed with HFD for 64 days	Liver: ACOT8, ACAD8	Liver: ACC, FAS	Lv Z et al. [165]	
	Buffalo Rat liver cells	–	–	5,10 μM for 24 h	pACC, CPT1, ACO	GPAT, FASN, CIDEC	(Zhong H et al., 2017) [244]	
	HepG2 cells	–	–	1, 10, 25 μM for 24 h	CPT1α	FASN, SCD1	Qin H et al. [166]	
EGCG	Wistar rats	–	I.G	50 mg/kg/d mixed with HFD for 100 wk	Liver: ACSL1, FABP1, CPT2	Liver: FASN, ACC1	Yuan H et al. [174]	Number↓ Accumulation↓
	SD rats	–	I.G	80 mg/kg/d for 30 days	–	Liver: pACC,FAS	Lin L et al. [177]	
	C57BL/6 J mice	–	P.O	50, 100 mg/kg mixed with HFD for 12wk	Subcutaneous Adipose: SCD1, ACC1, FAS, HSL, ATGL, ACO2, MCAD, PGC1α	Epididymal Adipose: ACC1, FAS, HSL, CPT1α, UCP2	Li F et al. [180]	
	Primary mouse hepatocytes	–	–	CCK8 assay with different doses for 48 h	SCD1	FASN	Yuanyuan Z et al., [242]	
CGA	SD rats	Orlistat	P.O	50, 100, 150 mg/kg b.w. mixed with HFD for 6 wk	Liver: CPT1	Liver: pACC	H VS et al. [185]	Number↓ Density↓ From spindle- shaped to round
	ICR mice	–	P.O	0.2 g/kg mixed with HFD for 8 wk	Liver: CPT1	Liver: FAS, ACAT	Cho AS et al. [186]	
	SD rats	–	I.G	60 mg/kg bw for 28 days	Liver: CPT1	Liver: FAS, ACC	Zhou Y et al. [187]	
	db/db mice	Metformin	I.G	0.25 g/kg/d bw for 18 days	Liver: CPT1a, ACOX1, ATGL HSL	Liver: MGAT1, CD36, DGAT1/2, FATP4	Yan Y et al. [189]	
	3T3-L1 cells	Rosiglitazone	–	20 μM for 10 days	HSL, DGAT1, PLIN	–	Peng SG et al. [191]	
	3T3-L1 adipocytes	–	–	50 μM for 24 h	FATP	–	Maetzin Becerra Sanchez et al. [240]	
	HepG2 cells	Simvastatin	–	30 μmol/L for 48 h	ABCA1	–	Shun Hao et al. [237]	
	Bovine mammary epithelial cells	–	–	40 μg/mL for 48 h	–	Lipin1	Ruiyuan Yao et al. [241]	
BET	Alcohol-fed C57BL/6 J mice	–	P.O	0.5% wt/vol for 5 wk	–	Epididymal adipose tissue: HSL	Dou X et al. [196]	Content↓ Accumulation↓ Uniform deposition of small LDs
	Ethanol-fed Wistar rats	–	P.O	1% wt/vol ethano for 6 mon	PGC-1α	Pancreas: FAS, DGAT1/2	Yang W et al. [195]	
	SD rats	–	P.O	62.5, 125, 250 mg/kg plus fructose for 5 wk	Liver: CPT1α	Liver: ADFP, CPTII, SCD1, FAS	(Ge CX et al., 2016)	
	SD rats	–	I.G	1 ml/d concentration of 400 mg/kg	Liver: CPT1	–	Xu L et al. [200]	
	Snout bream fingerlings	–	P.O	0.6, 1.2, 1.8% mixed with HFB for 8 wk	Liver: CPT1, MTTP	Liver: FAS	Adjoumani JY et al. [201]	
	OA-induced HepG2 cells	–	–	20, 40, 80, 160 mM for 24 h	ATGL	FASN, ACC	Chen W et al. [202]	
	Cells of Landes Goose fatty liver	–	–	20 mmol/L for 48 h	LPL, FABP	FAS, DGAT2	Liu J et al. [199]	
GIN	NAFLD SD rats	simvastatin	I.G	30, 60 mg/kg/day for 8 wk	Liver: CPT1, CPT2	–	Hou Y et al. [205]	Size↓ Sontent↓ Accumulation↓
	C57BL/6 J mice	–	I.G	20 mg/kg/d for 4 wk	–	Liver: C/EBP-α, FAS, aFABP, CD68, F4/80	Gu W et al. [214]	
	HFD C57BL/6 J mice	–	I.G	5, 10, 20 mg/kg for 3 wk	–	Liver: ACC, FAS, SCD1	Quan HY et al. [208]	
	C57BL/6 J mice	–	I.G	30, 100, 300 mg/kg for 5 days	Liver: HSL, CES1	Liver: FAS, SCD1	Li G et al. [209]	
	HFD KM mice	–	I.G	5, 10 mg/kg/d for 4 wk	WAT: HSL	WAT: C/EBPα, FAS, PLIN	Liu H et al. [215]	
	3T3-L1 cells	–	–	50, 100 μM for 2 days	–	C/EBP-α, FAS, aFABP, CD68, F4/80	Gu W et al. [214]	
	3T3-L1 cells	–	–	80 μM for 72 h		ACC, FAS, FABP4, PLIN	Liu H et al. [215]	
	HepG2 cells	–	–	1, 10, 50,100 μM for 8, 24, 48 h	–	ACC-α	Lee S et al. [213]	
	3T3-L1 adipocytes	–	–	20 μM for 6 days	PGC-1a, CPT1	–	Liu Z et al. [218]	
	3T3-L1 adipocytes	–	–	0, 10, 20, 30, 40 µM for 8 days	pACC	FABP4, C/EBPα	Oh JM et al. [210]	
	3T3-L1 cells	–	–	10, 50, 100 mM for 48 h	–	PLIN	Siraj FM et al. [217]	
RES	C57BL/6 J mice	Metformin	I.G	400 mg/kg mixed with DMSO for 45 days	Skeletal muscle: PLIN5	BAT, heart tissue: PLIN5	Mehdi F et al. [229]	Number↓ Size↓ Myocytes:number↑
	C57BL/6 J mice	–	P.O	0.2% combined with HFD for 8 wk	–	Liver: ADFP	Nishikawa K et al. [225]	
	C57BL/6 J mice	–	P.O	0.005%, 0.02% mixed with HFD for 6 wk	–	Epididymal WAT Liver: FAS, PAP	Cho SJ et al. [220]	
	HFD-induced C57BL/6 J mice	–	I.G	400 mg/kg/day for 30 days	–	Liver: FSP7β, ATF6, CREBH, PLIN1	Zhou R et al. [222]	
	HepG2 cells	–	–	5, 15, 45, 135 μmol/L plus OA for 48 h	pACC	Lipin	Tang LY et al. [223]	
	OA-induced primary hepatocytes	–	–	0. 20, 50, 100 μmol/L for 12 h	–	PLIN, Adipophilin	Wang C et al. [224]	
	FFA-induced L02 hepatocytes	–	–	20 μM for 24 h	–	FAS, ACC1, SCD1	Jing Y et al. [221]	
	Stromal vascular cells	–	–	50 μmol/L for 48 h	UCP-1, CIDEA, PGC-1a	–	Wang S et al. [219]	

### Berberine (BBR)

**Fig. 4:**
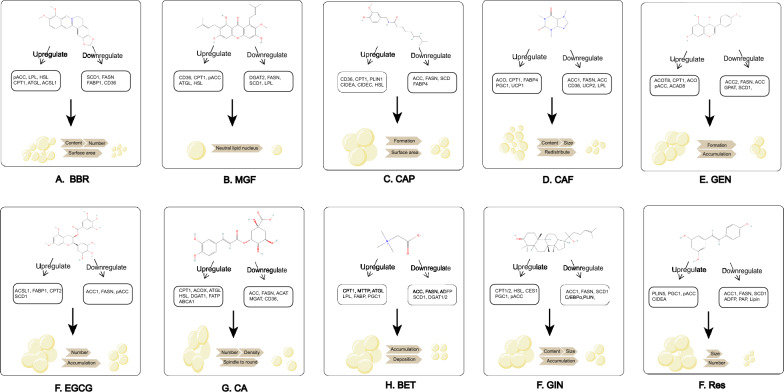
Chemical structures and mechanism of natural compounds. (**A**) BBR; (**B**) MGF; (**C**) CAP; (**D**) CAF; (**E**) GEN; (**F**) EGCG; (**G**) CGA; (**H**) BET; (**I**) GIN; (**J**) RES. All the chemical structures were obtained from pubchem: https://pubchem.ncbi.nlm.nih.gov/

BBR (Fig. 4A), is an isoquinoline alkaloid primarily enriched in the rhizomes of *Berberis spp*., *Coptis*, and cortex of *Phellodendron* [128]. BBR is originally used to treat diarrhea and has since been found to have a positive therapeutic effect on glycolipid metabolism disorders. BBR can attenuate hepatic TAG accumulation caused by overnutrition through reducing the expression of stearoyl coenzyme a desaturase 1 (SCD1) [129], fatty acid uptake-related proteins (e.g. FABP1, CD36), and fatty acid oxidation-related proteins (e.g. CPT1a) via the AMPK-SREBP1c pathway [130]. Wang, et al. found that BBR intake decreased liver fat content while elevating muscle lipid accumulation in black snapper fed with an HFD. The promotion of lipid mobilization by BBR may be related to the regulation of proteins associated with stearoyl coenzyme a desaturase (ACCα) HSL, and lipoprotein lipase (LPL) [131]. Based on the studies above, by restricting SCD1/CD36-related fatty acid synthesis and enhancing HSL/LPL-related lipolysis, BBR reduces LD content by restricting the enlargement of neutral fat nuclei.

Moreover, BBR has been reported to reduce the number and surface area of LDs in porcine oocytes at the IVM stage, stimulating miR-192 to downregulate SREBP1 and PPARγ [132]. Nevertheless, the detailed downstream effects of SREBP1 and PPARγ were not mentioned. Studies in colon cancer cells revealed that BBR inhibited lipogenesis and LD accumulation by promoting the ubiquitinated degradation of promyelocytic leukemia zinc finger-mediated SREBP-cleavage-activating protein [133]. The findings suggest that BBR may regulate LD through the PPARγ-SREBP1 pathway.

### Mangostin (MGF)

MGF is a botanical isoflavone (Fig. 4B), mainly existing in *Iris unguicularis*, *Mangifera indica*, *Cyclopia genitives*, *Salacia chinensis*, *Bombax ceiba* and *Anemarrhena asphodeloides *[134]. MGF is utilized to reduce serum TAG, FA, and improve blood lipid profiles [135]. Studies in NAFLD models have shown that MGF inhibited the reactivity of ACC, DGAT2, long-chain acyl-coenzyme A synthase 1 (LACS1), and SCD1 by decreasing SREBP1c and increasing pAMPK [136–138]. As a downstream effect of AMPK, changes in ACC should be elaborated more accurately, specifically the pACC to ACC ratio [139]. Moreover, the PPARα pathway was activated following MGF treatment, resulting in increased fatty acid translocase (FAT/CD36), HSL, ATGL, LPL, CPT1 [137, 138]. Consequently, LDs in HepG2 cells and C2C12 cells declined, and FA-induced IR reduced [140, 141]. MGF promotes CPT1-related β-oxidation, and inhibites enzymes related to TAG synthesis to reduce the content of neutral fat nuclei in LDs.

An in vitro model of AS indicated that MGF increased the expression of ATP-binding cassette A1/G1 (ABCA1/G1), PPARγ, and LXRα, promoted macrophage excretion and prevented lipid accumulation [142]. Interestingly, MGF reversed the expression of metabolically active CPT1 in human HT29 colon cells, suggesting that the regulation of fatty acid β-oxidation by MGF varies across models [143]. In addition, the metabolite of MGF, desmethylthiol, reduces the levels of TAG and FA in HepG2 cells by regulating the silent information regulator of transcription 1 (SIRT-1) /AMPK pathway [144]. As regulators of lipid autophagy, PPAR and LXR regulated by MGF increase autophagy and thus decrease the number of LDs.

### Capsaicin (CAP)

CAP (Fig. 4C), a phenolic compound derived from the genus Capsicum in the family *Solanaceae*, is a dietary agent that improves metabolism [145]. In adipocyte experiments, CAP can reduce the size and surface area of LDs by elevating pAMPK, LPL and HSL, PR structural domain containing 16 (PRDM16), CIDEA, PPARγ, uncoupling protein 1 (UCP1) [146–150]. Higher expression of pAMPK also inhibited the AKT/mTOR pathway, decreasing the basal neutrophil content in HepG2 cells [151]. The appliance of CAP in bovine bone marrow mesenchymal stem cells (BMSC) suggests that lipid deposition was inhibited during lipogenic differentiation with a lower transcription of PPARγ, FABP4 and SCD [152, 153].

The in vivo studies have demonstrated that CAP inhibits hepatic LD formation and increases plasma high density lipoprotein-C by activating transient receptor potential vanilloid 1, mediating PPAR, CPT1and CD36, and downregulating fatty acid production (e.g. ACC, FASN), which has implications for the treatment of AS and hypercholesterolemia [154–157]. It’s also worth noting that CAP cream can be given onto shaved abdominal skin, which provides a new approach for abdominal obesity. CAP mitigates LD accumulation by participating in all processes of lipid metabolism: inhibition of FABP4/SCD-related fatty acid synthesis, promotion of CPT1/CD36-related β-oxidation and lipolysis, and UCP1-mediated adipose tissue browning.

### Caffeine (CAF)

CAF (Fig. 4D) is a methylxanthine that appears widely in tea trees and coffee, and clinical studies demonstrated that its consumption has a negative correlation with the level of liver fibrosis [158]. CAF lowers the amount and volume of LDs in over-nourished zebrafish larvae and reduces LD levels in rat adipose-derived stem cells through upregulating CD36, ACOX and downregulating SREBP1c, SREBP2, ACC1, PPARγ, LPL, FASN, UCP2, and SCD1) [159, 160]. The promotion of adipogenic enzymes and inhibition of PPARα induced by HFD are reversed along with higher expression of hepatic CPT1 [161, 162]. CAF inhibits the adipogenic differentiation of fibroblasts by suppressing the transcription of CCAAT/enhancer-binding protein β (C/EBPβ), PPARγ, C/EBPα in Graves, ophthalmopathy [167, 168]. As a whole, ACC1, FASN, and SCD1 are adipogenesis-related proteins, while CD36 and CPT1 are lipogenesis or β-oxidation-related proteins. They are the main targets for CAF to reduce LDs.

In addition, CAF may induce LD autophagy in hepatocytes through rapamycin complex 1 and AMPK, but the exact regulatory mechanism needs further exploration [164]. VELICKOVIC, et al. found that CAF upregulated the browning genes (e.g. UCP1, PPARγ), PRDM16, and PGC-1α in mouse mesenchymal stem cells (MSCs), with LDs becoming smaller and redistributing evenly [163]. MSCs are an in vitro model of BAT, thus the effective concentration for in vivo application requires investigation [164].

### Genistein (GEN)

GEN (Fig. 4E), an isoflavone, is the major phytoestrogen in soybean. In a laying-hen FLD model, GEN inhibits the LD formation of hepatocytes [165, 166]. The expression of PPARα and its downstream ACOX, CPT1, and SREBP1c and its downstream GPAT, FASN, LXRα, ACC, and SCD1 is elevated. The expression ofFATP is downregulated [167]. Several in vitro studies also supported the ability of GEN to inhibit FA-induced LD formation in a dose-dependent manner [168].

The PPARγ-SREBP pathway may also play a role in the regulation of LDs by GEN. In the liver of non-obese rats, GEN can enhance SREBP2 and restrain SREBP1, FASN, PPARγ and its target gene CIDEC [169, 170]. It has been reported that GEN downregulate PPARγ and upregulate lipocalin by activation of estrogen receptor β and Akt/mTOR signaling, ameliorating hepatic fat accumulation [171, 172]. The downstream of pathways above requires further elaboration. CIDEC present in LD-mitochondrial contact promotes TAG transfer, while inhibiting the activity of ATGL. Lipid droplet-associated proteins, which undergo opposite alterations upon GEN intervention, are suspected to be due to differences in tissue specificity and induction models. Lipid droplet-associated proteins, which undergo opposite alterations upon GEN intervention, are suspected to be due to differences in tissue specificity and induction models. In conclusion, GEN restricts neutral fat nuclei growth by upregulating ACOX/CPT1-related lipolysis and downregulating ACC/CIDEC/SCD1-related lipogenesis.

### Epigallocatechin-3-gallate (EGCG)

EGCG (Fig. 4F), is the major polyphenol and catechin in green tea [173]. EGCG attenuates liver fibrosis, hepatic LDs, glomerular necrosis and FA levels in HFD-fed mice by inhibiting cholesterol synthesis through the SREBP2/SIRT1/FOXO1 pathway [174, 175]. The activation of SREBP2 and its target gene 3-hydroxy-3-methylglutaryl-coenzyme A reductase (HMGCR) enhance cholesterol synthesis [176]. The proteomics indicated a higher expression in FATP1, ACSL1, and CPT2; but a lower expression in SREBP2 and its downstream (ACC1, FASN) [177, 178]. An in vitro study demonstrated that EGCG promoted lysosomal autophagy through a Ca^2+^/CaMKKβ/AMPK-dependent mechanism in vascular endothelial cells [179].

Moreover, EGCG has tissue variability when coming into effect. EGCG significantly increases the expression of lipid synthesis in WAT while relevant genes in the epithelium tissue are downregulated [180]. In contrast to the control groups, there is a decline in the abdominal lipid rate, LPL, PPARγ and hepatic lipid synthase activity (e.g. FASN, ACC), as well as an increase in ATGL and CPT1 expression [181]. Proteins related to fatty acid transport and oxidation are increased while fatty acid synthesis-related proteins are reduced, resulting in fewer LDs.

### Chlorogenic acid (CGA)

CGA (Fig. 4G), a bioactive dietary polyphenol derived from tea and green coffee, can treat hepatic steatosis, cardiovascular diseases, and diabetes [182]. CGA can decrease the amount and density of LDs in liver by upregulating PPARα [183, 184]. Moreover, CGA increases pAMPK to suppress the expression of FASN, PPARγ2 and ACC [185–187]. Even so, Mubarak, A. et al. failed to favor the role of CGA in reducing lipid accumulation [188].

A study of diabetic mice showed that CGA had similar effects to metformin in reducing hepatic lipid levels by increasing ATGL, HSL, CPT1a, and ACOX1 and decreasing MGAT1, DGAT1, DGAT2, CD36, FATP4, and LPL) [189, 190]. FATP4 enlarges the core of LDs by mediating. Overall, CGA restricts LD growth by promoting ATGL-associated lipolysis and β-oxidation, and inhibiting of MGAT/DGAT/FATP-related synthesis and transport.

During 3T3-L1 preadipocyte differentiation, CGA downregulates the expression of adipocyte differentiation inhibitor gene and upregulates the expression of adipose transcription factors (e.g. CEBPB, SREBP1), differentiation-related transcription factors (e.g. PPARγ2), and lipogenic pathway-related genes (PLINs, SREBP1) [191]. Therefore, improved adipocyte differentiation changes LDs from spindle-shaped to round, while more HSL reduces intracellular TAG levels [192]. 3T3-L1 preadipocyte undergoes four differentiation stages, thus the effect of CGA in specific stages remains to be clarified [193].

### Betaine (BET)

Betaine (Fig. 4H), a trimethyl derivation of glycine, presents in beets, spinach, whole grains, wheat bran [194]. Studies in rats showed that BET diminished the level of LDs and swollen mitochondria in the liver [195]. The expression of DGAT1/2, SREBP1c/2, HSL, ACC and FASN genes is significantly reduced, except for the increased mRNA level of PGC-1α, PGC-1β [196]. In alcohol-induced pancreatic steatosis, BET also reverses pathological changes like LD accumulation and elevated lipocalin levels [197]. However, preclinical models exposed to alcoholic diet cannot summarize hallmarks of human alcohol-associated liver disease (ALD) due to the complicated pathogenesis of ALD [198].

In vitro, smaller LDs deposit uniformly after BET intervention [199]. The genes of SREBP1c, SIRT1, FASN, ACC DGAT2 are downregulated, but genes of MTP, LPL, PPARα, CPT1, ATGL are upregulated [200–202]. Similarly, suppression of fatty acid synthesis and deposition, promotion of oxidation and transport by BET maintain LD homeostasis.

### Ginsenoside (GIN)

GIN (Fig. 4I), the major active ingredient of *Chinese ginseng*, contains a group of triterpenoid saponins with different polarity (Rb1, Rg2, Rk3, Rk1, F2, etc.), which apply to IR and FA metabolism [203]. Rb1 promotes CD137, UCP1 [204], PPARα and its downstream (e.g. cholesterol 7 α-hydroxylase, CPT1α/2) in a dosage-dependent approach [205]. The activation of pAMPK (total horizontal of AMPK is unchanged) by Rb1 increases AS plaque stability by inhibiting SREBP, ACC-α, FASN, and SCD1 [206–208]. The pathways include activation of autophagic SIRT1/AMPK, PTEN/AKT, and inhibition of extracellular regulated protein kinases (ERK) /p3 and AKT [209, 210]. AKT intervenes the insulin signaling pathway, so natural compounds with insulin-like activities may regulate LDs [211].

In vitro, Rb1 downregulates the expression of FATP2, CD36, FABP1, FATP5, C/EBPβ and transcription factor PPARγ, and upregulates the expression of CPT1 and ACOX1 [212–216]. Siraj, et al. proved that F2 inhibited the mRNA levels of PLINs in adipocytes [217]. As a whole, GIN remarkably reduces LD diameter by promoting CPT1-mediated autophagy and browning-related genes, and inhibiting FA synthesis.

#### Resveratrol (RES)

RES (Fig. 4J), a dietary polyphenol from red wine and grapes, prevents AS and hepatic steatosis. RES reduces the number of LDs in adipocytes by increasing the expression levels of PGC-1α, UCP1, PRDM16 and CIDEA through the mTOR pathway, AMPK and ACC [218–220]. In FA-intervened human L02 hepatocytes, RES can downregulate the expression levels of SREBP1 and its target genes (e.g. SCD1, ACC1, and FAS) [221]. RES reverses the HFD-induced reduction in SIRT1 activity and elevation of—FSP27β/CIDEC, activating transcription factor 6 (ATF6), cyclic-AMP response binding protein H (CREBH), lipophilic, TIP-7 especially PLINs) [222–224]. Similarly, additional study found that RES could alleviate the expression of the adipose differentiation-related protein in the mouse liver through SIRT1/ATF6 signaling pathway [225]. SIRT1/AMPK also regulates mitochondrial autophagy [226, 227]. As a sensor of energy metabolism, SIRT1 regulates over 70 substrates like PGC-1α and Forkhead Box O (FOXO) by sensing the changes of Nicotinamide Adenine Dinucleotide (NAD^+^) levels [228]. Therefore, RES reduces LD accumulation by inhibiting FA synthesis and promoting adipose tissue browning.

A study on obesity found that RES attenuated the expression level of PLIN5 in BAT and heart tissue [229], while increasing the expression of PLIN5 in skeletal muscle. The clinical trial also manifested that the number of LDs in myocytes from T2DM patients increased after RES intake and LDs containing PLIN5 increased notably, but this change may help mitigate IR [230]. As a potential direct target of RES in the LD-mitochondrial contact, PLIN5 interacts with FATP, facilitating FA transfer.

## Discussion

In recent years, LDs has been acknowledged as an organelle whose size, number, morphology, and composition vary dynamically in response to an individual’s metabolic state. From a physiological standpoint, LD biogenesis serves to sequester detrimental free FAs, thereby mitigating ER stress and oxidative stress induced by lipotoxicity. Disruption of LD homeostasis can result in impaired or overloaded fatty acid storage, leading to lipotoxicity-related diseases like T2DM and fatty liver. Excessive accumulation or ectopic deposition of LDs is also closely related to the pathology of cardiovascular disease, chronic kidney disease, etc. To summarize, the significant role of LDs in chronic diseases associated with lipid metabolism has been confirmed. At the same time, LD-associated proteins have also gradually attracted significant attention. This class of proteins participants in LD biosynthesis and is also the direct executor of their biological functions. Targeting LD-associated proteins to regulate the “fate” of LDs may hold great promise for the development of innovative therapies. Given that LDs exhibit dynamic changes, their state varies in different diseases or at different stages of the same disease. As such, it is crucial to flexibly regulate LD homeostasis and biological functions.

Drugs targeting LD-associated proteins are already in clinical use. DGAT2 inhibitors and ACC inhibitors can reduce liver fibrosis [231]. ACAT1 inhibitors disrupt the biogenesis of CE-rich LDs, reducing cancer proliferation and aggressiveness in prostate cancer [232]. Nonetheless, these specific inhibitors have the potential to cause harm to other regular cellular metabolic processes [54].

**Fig. 5:**
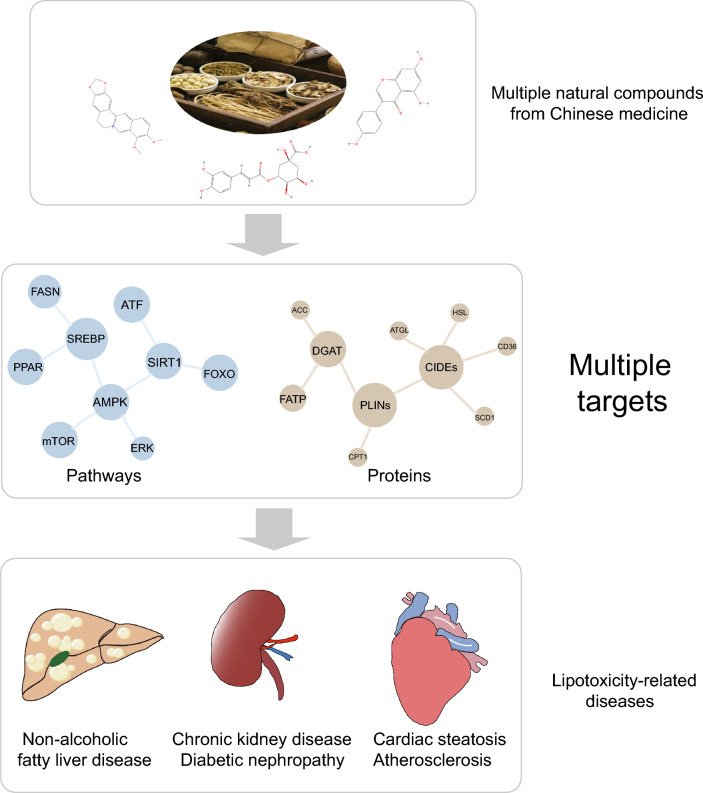
The effects of natural compounds on lipotoxicity-related metabolic diseases from the perspective of LD-associated proteins

Natural compounds have positive prospects in the treatment of metabolic diseases due to their safety, effectiveness and multiple targets. There are numerous natural compounds with lipid-regulating, glucose-lowering, and oxidative stress-reducing effects. However, their pharmacological mechanisms are not been fully understood, limiting their potential for clinical use. We outlined the mechanism of 10 common natural compounds, focusing on the LD-associated proteins and pathways they regulate. We emphasize that LDs may be a target organelle of natural compounds, and LD-associated proteins may serve as important targets of these compounds or downstream effectors or upstream regulators of pathways (Fig. 5).

Despite their potential, research on natural compounds related to LD-associated proteins remains restricted:

1. Most studies are in vivo and in vitro studies, with a lack of clinical data.
2. Only phenotypic improvement and regulatory pathway data are presented, without in-depth analyses like protein interaction.
3. Limited focus is placed on LD-associated proteins, concentrating on PLINs and CIDEs.
4. Attention is mainly on LD in the liver or adipose, with less focus on cardiovascular, cerebrovascular, kidney and other organs.

Future studies should employ drug-target identification methods to focus more closely on the direct targets of natural compounds, specifically LD-associated proteins. The specific effects of natural compounds on LD-associated proteins, including post-translational modifications and protein interactions, remain unclear. As LD is a dynamically changing organelle, it is essential to understand how natural compounds regulate LD biology in different diseases or stages of the same disease. Frequent contact between LDs and other organelles can affect LD homeostasis, so it is important to investigate whether natural compounds mediate this contact.

Natural compounds also face challenges such as rapid metabolism, insufficient absorption, and poor solubility. For example, CAP has a significant first pass metabolism and a very short half-life by intravenous administration [233, 234]; BBR has poor solubility with less than 1% of oral bioavailability [235]. These drawbacks result in limitations in clinical use and dissemination [236]. As a result, the development of metabolites or derivatives from natural compounds with higher bioavailability may facilitate their clinical applications.

## Conclusion

This review has summarized the current understanding of LDs, LD-associated proteins, and pathological changes arising from abnormal LD metabolism. Moreover, we provide the first overview of the therapeutic mechanisms of natural compounds with lipid-modulating effects in metabolic diseases from the perspective of LDs and LD-associated proteins.

Previous research has shown that natural compounds positively impact metabolic diseases by regulating LD homeostasis. The underlying mechanisms may be closely related to biological processes such as LD biogenesis, growth, fusion, and degradation mediated by LD-associated proteins including PLINs, CIDEs, neutral lipid synthases, lipolytic enzymes, and so on. LD deserves extensive attention as a target organelle for natural compounds. Given that natural compounds exhibit a wide range of biological activities, their therapeutic effects may also involve multiple signaling pathways. This complexity makes it difficult to elucidate whether LD-associated proteins can be direct targets of action for natural compounds with the available evidence. As a novel research target for this class of drugs, there is a need to accurately identify more LD-associated proteins, and provide more reliable and direct evidence of drug-target interactions. Challenges remain to improve the bioavailability of natural compounds and to conduct relevant clinical trials. All in all, future studies on LD and LD-associated proteins will hold promising prospects for the development and utilization of natural compounds.

## Data Availability

Not applicable.

## Abbreviations

LD: Lipid droplet
NAFLD: Non-alcoholic fatty liver disease
PLIN: Perilipin
CIDE: Cell death-induced DNA fragmentation factor 45-like effector
HIG2: Hypoxia inducible gene 2
TAG: Triacylglycerol
CE: Cholesterol ester
FA: Fatty acid
DAG: Diacylglycerol
ER: Endoplasmic reticulum
LDAF1: LD assembly factor 1
AGPAT2: 1-Acylglycerol-3-phosphate O-acyltransferase 2
Lipin-1: Lipoprotein 1
PA: Palmitic acid
GPAT: Glycerol-3-phosphate acyltransferase
nLD: Nucleus LD
cLD: Cytoplasmic LD
PC: Phosphatidylcholine
PCYT: Phosphocholine cytidylyltransferase
DGAT2: Diacylglycerol acyltransferase 2
PAP: Phosphatidic acid phosphatase
MFN2: Mitofusin2
HSC70: Heat shock cognate 71 kDa protein
ARFRP1: ADP-ribosylation factor related protein 1
SNAP23: Synaptosome-associated protein 23
MIGA2: Mitoguardin 2
VPS13D: Vacuolar Protein Sorting 13D
UBXD8: UBX structural domain-containing protein 8
CGI-58: Comparative gene identification-58
ATGL: Adipose triglyceride lipase
PNPLA2: Patatin-like phospholipase domain-containing protein 2
WAT: White adipose tissue
HSL: Hormone-sensitive triglyceride lipase
MAGL: Monoacylglycerol lipase
SREBP: Sterol regulatory element binding protein
LXR: Liver X receptor
PPAR: Peroxisome proliferators-activated receptor
CMA: Chaperone-mediated autophagy
HOPS: Homotypic fusion and protein sorting
SNARE: Soluble NSF attachment protein receptor
FASN: Fatty acid synthase
HFD: High-fat-diet
AMPK: Adenosine 5'-monophosphate-activated protein kinase
VLDL: Very low-density lipoprotein
T2DM: Type 2 diabetes mellitus
1-MAG: 1-Monoacylglycerols
STX1a: Synthesis protein 1a
CKD: Chronic kidney disease
CPT1: Carnitine palmitoyltransferases 1
ACOX1: Acyl-Coenzyme A oxidase 1
L-FABP: L-fatty acid binding protein
LDLR: LDL receptor
DN: Diabetic nephropathy
AKI: Acute kidney injury
PGC-1α: PPARγ coactivator-1α
AS: Atherosclerosis
ACAT1: Acyl coenzyme A-cholesterol acyltransferase
IRI: Ischemia–reperfusion injury
ACSL: Acyl-CoA synthetase long chain family member
CGI: Congenital lipodystrophy
IR: Insulin resistance
mTOR: Mammalian target of rapamycin;
BBR: Berberine;
SCD: Stearoyl coenzyme a desaturase
ACCα: Acetyl coenzyme carboxylase α
LPL: Lipoprotein lipase
MGF: Mangostin
LACS1: Long-chain acyl-coenzyme A synthase 1
ABCA1/G1: ATP-binding cassette A1/G1
SIRT1: Silent information regulator of transcription 1
PRDM16: PR structural domain containing 16
UCP1: Uncoupling protein 1
BMSC: Bone marrow mesenchymal stem cell
MSCs: Mesenchymal stem cells
C/EBPβ: CCAAT/enhancer-binding protein β
GEN: Genistein
FATP: Fatty acid transporter protein
EGCG: Epigallocatechin-3-gallate
HMGCR: 3-Hydroxy-3-methylglutaryl-coenzyme A reductase
CGA: Chlorogenic acid
ALD: Alcohol-associated liver disease
ERK: Extracellular regulated protein kinases
RES: Resveratrol
ATF: Activating transcription factor
CREBH: Cyclic-AMP response binding protein H
FOXO: Forkhead Box O
NAD^+^: Nicotinamide adenine dinucleotide
CAP: Capsaicin
CAF: Caffeine
BET: Betaine
GIN: Ginsenoside
